# Efficacy of Acupuncture in the Treatment of Essential Hypertension: An Overview of Systematic Reviews and Meta-Analyses

**DOI:** 10.1155/2023/2722727

**Published:** 2023-04-18

**Authors:** Maoxia Fan, Guohua Dai, Runmin Li, Xiaoqi Wu

**Affiliations:** ^1^Shandong University of Traditional Chinese Medicine, Jinan, Shandong Province, China; ^2^Affiliated Hospital of Shandong University of Traditional Chinese Medicine, Jinan, Shandong Province, China

## Abstract

**Background:**

Acupuncture is widely used in the clinical treatment of essential hypertension (EH). This overview is aimed at summarizing current systematic reviews of acupuncture for EH and assessing the methodological bias and quality of evidence.

**Methods:**

Two researchers searched and extracted 7 databases for systematic reviews (SRs)/meta-analyses (MAs) and independently assessed the methodological quality, risk of bias, reporting quality, and quality of evidence of randomized controlled trials (RCTs) included in the SRs/MAs. Tools used included the measurement tool to assess systematic reviews 2 (AMSTAR-2), the risk of bias in systematic (ROBIS) scale, the checklist of preferred reporting items for systematic reviews and meta-analyses (PRISMA), and the grading of recommendations assessment, development, and evaluation (GRADE) system.

**Results:**

This overview included 14 SRs/MAs that use quantitative calculations to comprehensively assess the various effects of acupuncture in essential hypertension interventions. The methodological quality, reporting quality, risk of bias, and quality of evidence for outcome measures of SRs/MAs were all unsatisfactory. According to the results of the AMSTAR-2 assessment, all SRs/MAs were of low or very low quality. According to the results of the ROBIS evaluation, a few SRs/MAs were assessed as low risk of bias. According to the results of the PRISMA checklist assessment, SRs/MAs that were not fully reported on the checklist accounted for the majority. According to the GRADE system, 86 outcomes were assessed under different interventions in SRs/MAs, and 2 were rated as moderate-quality evidence, 23 as low-quality evidence, and 61 as very low-quality evidence. Limitations of the included SRs/MAs included the lack of necessary items, such as not being registered in the protocol, not providing a list of excluded studies, and not analyzing and addressing the risk of bias.

**Conclusion:**

Currently, acupuncture may be an effective and safe treatment for EH, but the quality of evidence is low, and caution should be exercised when applying this evidence in clinical practice.

## 1. Introduction

Essential hypertension (EH) is a common clinical disease caused by multiple factors such as heredity and environment. The main clinical manifestation is elevated arterial pressure in the systemic circulation, which can cause serious damage to multiple organs such as blood vessels, the heart, brain, kidneys, and eyes [1]. There is a strong, independent linear association between blood pressure and cardiovascular disease risk. Hypertension is an independent risk factor for cardiovascular disease [2], and the concurrent cardiovascular and cerebrovascular diseases not only cause disability and high mortality but also consume medical and social resources, causing a heavy burden on families, society, and the health system [3]. With the increase of life and work pressure, the incidence of hypertension is increasing year by year, and the affected population is getting younger and younger. Against this background, the cardiovascular disease has become a heavy burden in my country, and effective control of hypertension is the top priority to relieve the burden of cardiovascular and cerebrovascular diseases [4]. Usually, the conventional treatment of EH is mainly western medicine, characterized by the initial use of a single antihypertensive drug, followed by dose titration and sequential addition of other drugs to achieve antihypertensive goals [5]. However, problems such as adverse reactions caused by long-term medication, drug tolerance, and high medical expenses have been increasingly concerned by patients. Therefore, it is imminent to find a treatment method that can effectively lower blood pressure, reduce adverse reactions, and effectively control complications [6].

Therefore, more and more scholars are exploring safer EH treatment methods. At present, some scholars have applied acupuncture with traditional Chinese medicine (TCM) to the treatment of EH. Many clinical studies [7–21] have shown that acupuncture can effectively control blood pressure; acupuncture may regulate the central nervous system [7–9] and the renin-angiotensin-aldosterone system (RAAS) [10–12], balance the imbalanced immune system [13–15], improve vascular structure [16–18], and reduce oxidative stress [19–21]. Therefore, it is believed that acupuncture may be a safer and more effective antihypertensive therapy.

In recent years, evidence-based medicine has been deeply integrated with TCM clinical research, especially acupuncture clinical research. From the perspective of evidence-based clinical practice, systematic reviews (SRs) should be the best evidence to guide clinical practice. Through preliminary searches, the team found that there have been multiple SRs showing that acupuncture therapy has a certain effect on EH, but the methodological quality of these SRs is unclear, and the quality of the evidence provided is uncertain. Therefore, our study aims to critically evaluate the methodological quality of SRs/MAs in the treatment of EH with acupuncture through a comprehensive overview to provide clinical ideas and evidence support.

## 2. Materials and Methods

The methodology of this overview follows the Cochrane Handbook, and the report of this overview is in line with the preferred reporting items for systematic reviews and meta-analyses (PRISMA) 2020 checklist [22]. This overview has been registered with the PROSPERO website (registration number: PROSPERO CRD42022361514).

### 2.1. Inclusion and Exclusion Criteria

The inclusion criteria are as follows: (1) SRs/MAs based on randomized clinical trials(RCTs), the language is limited to Chinese and English; (2) a definite diagnosis of EH, regardless of type, gender, age, and course of disease; (3) interventions in the treatment group included one of the three methods of simple acupuncture, electroacupuncture, and warm acupuncture or one of the abovementioned three methods combined with the treatment recommended by the guidelines (including lifestyle regulation and conventional antihypertensive drugs (CAD)), while the control group received a guideline-recommended treatment or a placebo/sham acupuncture; (4) the main outcome indicators were the effective rate of blood pressure reduction, the improvement of systolic blood pressure (SBP) and diastolic blood pressure (DBP), the change of blood pressure before and after treatment, and adverse reactions.

The exclusion criteria are as follows: (1) duplicate publications, animal studies, research protocols, narrative reviews, reviews, and network meta-analysis; (2) works of literature with incomplete data or unidentifiable original text; (3) the subjects of the study were EH combined with other illnesses.

### 2.2. Search Strategy

A systematic search was conducted through PubMed, Cochrane Library, Embase, Web of Science, the Chinese National Knowledge Infrastructure (CNKI), Chinese Biological Medicine Database (CBM), and Wanfang Database; the search period is from the inception of the database to September 30, 2022, and hand-searched and traced references were adopted to supplement relevant literature. The search used a combination of subject words and free words. Key search terms were derived from MeSH and included search terms such as: “Acupuncture,” “Essential hypertension,” “Systematic review,” OR “meta-analysis”. The search strategies used in PubMed are shown in Table 1. The adjustment was made to suit the specific needs of each database when necessary. In addition, we searched conference proceedings and dissertations to identify relevant gray literature.

### 2.3. Literature Screening and Data Extraction

Two researchers (MX-F and XQ-W) independently screened and extracted the literature and cross-checked them. The disagreement (if any) was discussed and negotiated or subjected to the decision of a third author (GH-D). The extracted literature information included authors, publication year, nationality, sample size, intervention measures, quality assessment tools, and main conclusions.

### 2.4. Quality Assessment

Two researchers (MX-F and XQ-W) independently assessed the methodological and evidence quality of the included SRs/MAs; any discrepancies were resolved by consensus or adjudication by a third author (GH-D).

#### 2.4.1. Methodological Quality Assessment

In 2007, clinical epidemiology and evidence-based medicine experts from research institutions in Netherlands and Canada developed and published a measurement tool to assess systematic reviews (AMSTAR). In the following 10 years, AMSTAR became an internationally recognized and most widely used evaluation tool. Subsequently, a large number of relevant documents using AMSTAR tools appeared. Research shows that the use of AMSTAR has played a positive role in standardizing the production and reporting of SRs/MAs and promoting the generation and dissemination of high-level evidence. A measurement tool to assess systematic reviews 2 (AMSTAR-2) [23] is a quality evaluation tool of SRs/MAs methodology newly developed on the basis of the first version of AMSTAR, with good consistency and practicality among evaluators.

AMSTAR-2 contains 16 items that can be answered with a “yes,” “partially yes,” or “no.” According to the evaluation criteria, it can be rated as “high,” “moderate,” “low,” and “very low,” and 7 out of 16 items in the tool (2, 4, 7, 9, 11, 13, and 15) are critical items.

#### 2.4.2. Risk of Bias Assessment

The risk of bias in systematic review (ROBIS) [24], which aims at the bias risk of system evaluation, is used not only to evaluate the bias risk in the process of producing and interpreting the results of multiple SRs/MAs such as intervention, diagnosis, etiology, and prognosis but also to evaluate the correlation between the SRs/MAs problems and the practical problems to be solved by users.

ROBIS is useful for assessing the extent of bias in four domains: (1) eligibility criteria for each study; (2) the identification and selection of studies; (3) data collection and study appraisal; and (4) overall synthesis and major findings. Within each domain, specific questions were used to determine the risk of bias, which was rated as “low,” “high,” or “unclear.”

#### 2.4.3. Report Quality Assessment

SR/MA is an important evidence to guide clinical practice. The clarity of its report affects the realization of its clinical value. Standard reports can reduce the bias between actual research results and published results and increase the transparency of articles. The PRISMA report guide is designed to help authors improve the quality of their reports, obtain key information, and improve readability and credibility.

The quality of each SR/MA report for the included SRs/MAs was assessed by the PRISMA 2020 [22] checklist, and each of the 27 items included in PRISMA 2020 was scored as “yes,” “partially yes,” or “no.”

#### 2.4.4. Evidence Quality Assessment

In order to be useful to decision-makers, clinicians, and patients, the SRs/MAs must provide not only the effect estimates of each result but also the information needed to judge the correctness of these effect estimates. The grading of recommendation assessment, development, and evaluation (GRADE) [25] provides a structure for SRs/MAs and clinical practice guidelines to ensure that it addresses all key issues of outcome evidence quality evaluation related to a specific issue in a consistent and systematic manner.

The quality of evidence for each SR/MA outcome was evaluated by the GRADE system. Since the initial quality of evidence for RCTs is high, the quality of evidence for the outcomes of the study was evaluated based on downgrading factors such as limitations, inconsistencies, indirectness, imprecision, and publication bias of the study. According to the downgrading level, they were rated as “high,” “moderate,” “low,” and “very low.”

## 3. Results

### 3.1. Literature Search and Screening Results

**Figure 1:**
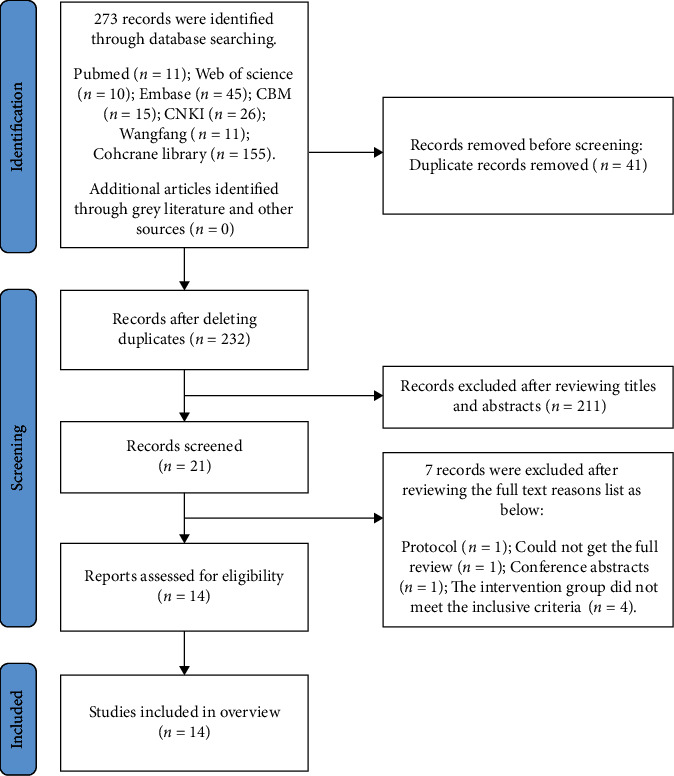
Flow diagram of the literature screening process.

A total of 273 works of related literature were retrieved, and 14 works of literature [26–39] were finally included after the layer-by-layer screening. The specific screening process is shown in Figure 1.

### 3.2. The Basic Characteristics of the Included Literature

Among the 14 SRs/MAs included, 5 [35–39] SRs/MAs were in English, 9 [26–34] SRs/MAs were in Chinese, 13 SRs/MAs were conducted in China, and 1 [34] SR/MA was carried out in South Korea, the publication year was 2009-2022, and the number of RCT studies ranged from 4 to 53. In terms of quality assessment of included RCTs, 8 SRs/MAs [28–30, 32, 34, 37–39] were assessed by the Cochrane risk of bias tool, and 5 SRs/MAs [26, 27, 31, 33, 36] were assessed by the Jadad scale, and 1 SR/MA [35] used the Oxford scale. The intervention measures in the treatment group were one of the three methods of simple acupuncture, electroacupuncture, and warm acupuncture or one of the above-mentioned methods combined with the relevant treatment recommended by the guidelines (including lifestyle regulation and CAD), while the control group received relevant treatments recommended by the guidelines or placebo/sham acupuncture. See Table 2 for specific information.

### 3.3. Results on SRs/MAs Quality Assessment

#### 3.3.1. Results of the Methodological Quality

The quality of the included SRs/MAs was assessed by AMSTAR-2, and the results showed that 12 SRs/MAs [26–37] were of very low quality because none of the included SRs/MAs met the criteria of item 2 (none of the included SRs/MAs has a registered protocol); 2 SRs/MAs [38, 39] were of low quality, and none of the 14 SRs/MAs met the criteria of item 7 (none of the SRs/MAs provided an exclusion list) and item 3 (no explanation for selecting the type of systematic review included in the study design). The methodological quality limitation also included the following items: item 1 (1 SR/MA author did not fully describe the PICO elements in the SR), item 8 (authors of 2 SRs/MAs did not fully describe essential characteristics of included studies), item 10 (2 SRs/MAs did not report funding RCTs/SRs/MAs), item 12 (authors of 12 SRs/MAs did not investigate the presence of risk of bias on the total effect), item 13 (authors of 4 SRs/MAs did not discuss the effect of risk of bias on the total effect of included studies), item 14 (authors of 8 SRs/MAs did not investigate sources of heterogeneity in results and/or did not discuss their effect on study results), item 15 (authors of 7 SRs/MAs did not test for publication bias and/or discussed its effect on results), item 16 (authors of 2 SRs/MAs did not describe funding sources and/or statements conflict of interest). The results are summarized in Table 3.

#### 3.3.2. Results of the Risk of Bias Assessment

Regarding the results of the ROBIS assessment, both phase 1 and domain 1 of phase 2 rated SRs/MAs as having a low risk of bias. In the phase 2, 4 SRs/MAs in domain 2 were rated as low risk of bias, 8 SRs/MAs in domain 3 were rated as low risk of bias, 1 SR/MA in domain 4 was rated as low risk of bias, and all SRs/MAs were rated as high risk of bias in phase 3. The ROBIS scale evaluation results are shown in Table 4.

However, there were also some reporting flaws in other projects. The reports for item 5 (methods: protocol and registration), item 8 (methods: search), item 15 (risk of bias across studies), and item 24 (funding) were incomplete (“Yes or Partial Yes” with a response rate less than 50%). The results of the PRISMA checklist assessment are shown in Table 5.

#### 3.3.3. Results of the Quality of the Evidence

Meta-analysis was performed on the outcome indicators in the study, and the GRADE system was used to evaluate the quality of 86 outcome indicators under different intervention indicators one by one. Of these, 2 were of moderate quality, 23 were of low quality, and 61 were of very low quality. Limitation downgrading due to the risk of bias was more common in included studies (*n* = 86), followed by imprecision (*n* = 63), publication bias (*n* = 62), inconsistency (*n* = 43), and indirectness (*n* = 0). See Table 6 for details.

#### 3.3.4. Summary of Results

The outcome measures extracted from the included studies are listed in Table 6.

*(1) The Effective Rate in Lowering Blood Pressure*. 6 SRs/MAs [26, 27, 30–32, 38] reported the effective rate of blood pressure reduction. 2 SRs/MAs [26, 27] reported that acupuncture alone was more effective than CAD in the treatment of EH; 4 SRs/MAs [30–32, 38] reported that acupuncture combined with CAD was more effective than CAD alone in the treatment of EH.

*(2) The Efficacy of Improving SBP and DBP*. 11 SRs/MAs [27, 29–34, 36–39] reported the efficacy of acupuncture in improving SBP and DBP. 9 SRs/MAs [27, 29, 31–33, 36–39] reported that acupuncture alone was more effective in treating EH than CAD or placebo/sham acupuncture or no treatment. 10 SRs/MAs [29–34, 36–39] reported that acupuncture combined with lifestyle regulation or CAD was more effective than lifestyle regulation or CAD or placebo/sham acupuncture in the treatment of EH

*(3) The Effect of Reducing the Magnitude of SBP and DBP*. 3 SRs/MAs [28, 35, 37] reported the magnitude of blood pressure reduction of SBP and DBP. 3 SRs/MAs [28, 35, 37] reported that acupuncture alone was more effective than CAD or placebo/sham acupuncture in the treatment of EH, and the efficacy of acupuncture combined with CAD in the treatment of EH was better than that of CAD alone or placebo/sham acupuncture combined with CAD

*(4) The Effective Rate of Comprehensive Treatment*. 3 [32–34] SRs/MAs reported the effective rate of comprehensive treatment. 2 SRs/MAs [32, 33] reported that the efficacy of acupuncture alone in the treatment of EH was better than that of CAD, and acupuncture combined with CAD or behavioral adjustment was better than CAD alone in the treatment of EH. 1 SR/MA [34] reported that acupuncture combined with TCM decoction Tianma Gouteng decoction (TMGTD) was more effective than CAD or TMGTD in the treatment of EH.

*(5) The Effective Rate of Improving Symptoms*. 2 SRs/MAs [26, 32] reported an effective rate of symptom improvement. 2SRs/MAs [26, 32] reported that the efficacy of acupuncture alone in the treatment of EH was better than that of CAD, and the efficacy of acupuncture combined with CAD in the treatment of EH was better than that of CAD alone

*(6) Adverse Reactions*. 8 SRs/MAs [28, 31, 32, 35–39] reported adverse reactions. The main adverse reactions included occasional acupuncture site bleeding, dizziness, headache, cough, nausea, and pain, which recovered spontaneously after rest and did not require treatment. Since most studies failed to report adverse reactions in a standard way, quantitative analysis was not performed.

## 4. Discussion

At present, the main treatment strategy for EH is to give CAD on the basis of lifestyle control combined with the risk assessment of hypertension. However, due to the problems such as drug tolerance and side effects, clinicians are paying increasing attention to the treatment of hypertension by TCM, especially acupuncture. At present, there has been some clinical evidence for acupuncture treatment of hypertension. We systematically searched the existing systematic reviews and meta-analyses and comprehensively analyzed the existing evidence. The main findings of the study are as follows.

This study is the first overview of systematic reviews of acupuncture for essential hypertension based on RCTs published between 2009 and 2022. Growing evidence suggests that acupuncture can be used as an adjunctive treatment for EH and reduce dependence on CAD. Overall, available evidence suggests that acupuncture alone or a combination of acupuncture and medicine is more effective than placebo (sham acupuncture) or conventional antihypertensive regimens in the treatment of EH. In terms of safety, acupuncture has no serious adverse reactions.

### 4.1. The Methodology Is Not Standardized

The results of the AMSTAR-2 evaluation showed that the methodological quality of the included works of literature was rated as very low or low, mainly due to the following problems:

(1) *Missing Preliminary Design Protocol:* The researcher should specify the preliminary design protocol in the SRs/MAs. Most of the SRs/MAs included in this article were not registered, which may have increased the risk of bias in the preparation of the systematic reviews.

(2) *Incomplete Literature Search:* The involved researchers searched at least two databases, but most of them did not conduct supplementary search and gray literature search

(3) *Failure to Provide a List of Excluded Literature:* It may affect the authenticity of the results. During the systematic review process, it is an integral part of high-quality SR/MA to provide a list of potentially relevant studies that did not meet the inclusion criteria and explain the reasons for the exclusion

(4) *Risk of Bias Analysis Assessment Flaws:* When RCTs of varying qualities are included, the authors should assess their impact on the study results through subgroup analysis, regression analysis, sensitivity analysis, etc. Some of the included systematic reviews have insufficient or no description of the risk of bias assessment, and the lack of assessment of publication bias may undermine the authenticity of the conclusions. Furthermore, if SRs/MAs do not report funding resources of the included RCTs, this may increase clinical trial bias, as findings from industry-funded studies may be biased in favor of the funders.

In the GRADE system rating, acupuncture has certain efficacy in the treatment of EH, but the quality of evidence is low. The reason lies in that the methodological quality of the 14 included SRs/MAs has certain defects and the methodological quality of the original studies included in each SR/MA is not high, which affects the strength of the demonstration of the outcome indicators. It also shows that the original research design of the current acupuncture therapy for EH has certain defects, lacking scientific and standardized methodological guidance; the sample size of the research is relatively small, which ultimately affects the potency of the systematic review. According to the graded evidence quality assessment, 2 of the 86 effect sizes were of moderate quality, 23 were of low quality, and 61 were of very low quality. Risk of bias was the most common downgrading factor, followed by imprecision, publication bias, inconsistency, and indirectness. The risk of bias is mainly reflected in the fact that most of the original RCTs on acupuncture for EH did not clearly describe random sequence generation, allocation concealment, or blinding. Through further analysis, the outcome measures included in the SRs/MAs were found to be at risk of publication bias. It is worth noting that although almost all SRs/MAs indicated that acupuncture is an effective treatment, the conclusions of SRs/MAs may differ from real-world results and require further confirmation in the future due to low methodological quality studies.

### 4.2. Implications for Future Research

To reduce biases in such aspects as selectivity, implementation, and measurement, further large-sample, multicenter, long-term clinical RCTs based on evidence-based medicine standards are needed in the original research, and attention should be paid to the correct and reasonable implementation of randomization, allocation scheme concealment and blinding, etc. In addition, to further improve the quality of the evidence, authors should pay attention to the registration of the study protocol to ensure the rigor of its procedures before conducting SRs/MAs studies. During literature retrieval and screening, the information of literature exclusion and the complete retrieval strategy of all databases should be listed and elaborated to ensure that the process can be reproduced by others. In the quantitative calculation of effect sizes, care should be taken to exclude the results of individual studies on a case-by-case basis to ensure the stability of the results. In addition, a comprehensive assessment of publication bias will also improve the accuracy of meta-analysis results.

### 4.3. Strengths and Limitations

This is the first overview that makes use of the above assessment tools to assess SRs/MAs regarding acupuncture for EH. On one hand, the evaluation results strengthen the quality of the current relevant SRs/MAs evidence; on the other, the evaluation process of AMSTAR-2, ROBIS, PRISMA, and GRADE grading revealed obvious limitations of SRs/MAs and RCTs, which may help guide future high-quality clinical research. However, we must also acknowledge the limitations of this overview. Due to language limitations, this study only included systematic reviews published in both Chinese and English and did not include Korean and Japanese databases with the same background in traditional Chinese medicine research. Besides, the retrieval process has actually omitted manual retrieval, resulting in selection bias to a certain extent. In addition, two researchers conducted literature screening and quality assessment, and the process was subjective, the quantity of SRs/MAs included was small, and the overall quality was not high.

## 5. Conclusion

At present, acupuncture has a certain curative effect in the treatment of EH, but the quality of the evidence is low. The evidence should be used with caution in clinical practice, where the actual situation should be fully considered and the application should be combined with the patient's value preference and economic factors.

## Figures and Tables

**Table 1:** Search strategy for the PubMed database.

Query	Search terms
#1	“Acupuncture” [mesh]
#2	“Acupuncture therapy” OR “needle therapy” OR “needle warming therapy” OR “electroacupuncture” OR “needle” OR “pinprick” OR “acupoint”
#3	#1 OR #2
#4	“Hypertension”[mesh]
#5	“Essential hypertension” OR “high blood pressure” OR “blood pressure”
#6	#4 OR #5
#7	Meta-analysis as topic [mesh]
#8	“Systematic review” OR “meta-analysis” OR “meta analysis” OR “meta-analyses” OR “review, systematic” OR “systematic reviews”
#9	#7 OR #8
#10	#3 AND #6 AND #9

**Table 2:** Basic information of the included SRs/MAs.

Author, year (country)	Language of publication	Trials (subjects)	Intervention group	Control group	Quality assessment	Main results
Liu et al., 2012 (China) [26]	Chinese	10 (782)	Acupuncture/acupuncture+antihypertensive drugs	Antihypertensive drugs	Jadad scale	Acupuncture is safe and effective in the treatment of mild to moderate essential hypertension.
Yu et al., 2013 (China) [27]	Chinese	18 (1462)	Acupuncture	Antihypertensive drugs	Jadad scale	The data indicate that acupuncture and drugs have the same therapeutic effect on essential hypertension.
Zhang et al., 2013 (China) [28]	Chinese	11 (1072)	Acupuncture/acupuncture+antihypertensive drugs	Sham acupuncture/antihypertensive drugs	Cochrane criteria	Acupuncture can reduce blood pressure to varying degrees without adverse reactions,but there are certain defects in the quality of the included studies, and more high-quality studies are needed.
Qian, 2013 (China) [29]	Chinese	18 (1473)	Acupuncture/acupuncture+conventional therapy	Sham acupuncture/antihypertensive drugs/conventional therapy	Cochrane criteria	Acupuncture has potential efficacy in the treatment of essential hypertension without serious adverse reactions
Guo et al., 2013 (China) [30]	Chinese	10 (679)	Acupuncture+antihypertensive drugs	Antihypertensive drugs	Cochrane criteria	The combination of acupuncture and medicine has good clinical efficacy in the treatment of essential hypertension.
Zhang et al., 2014 (China) [31]	Chinese	13 (1066)	Acupuncture/acupuncture+antihypertensive drugs	Antihypertensive drugs	Jadad scale	Acupuncture is a valuable medical method, and to a certain extent, it can be used as a safe, green, and beneficial therapy to replace western medicine for lowering blood pressure, and acupuncture combined with antihypertensive has better curative effect.
Zhang et al., 2017 (China) [32]	Chinese	53 (4459)	Acupuncture/acupuncture+antihypertensive drugs	Antihypertensive drugs	Cochrane criteria	A comparison of acupuncture/acupuncture combined with antihypertensive drugs and pure antihypertensive drugs has a good effect in improving all aspects of the condition of hypertensive patients.
Zhu and Ding, 2018 (China) [33]	Chinese	22 (1758)	Acupuncture/acupuncture+antihypertensive drugs/acupuncture+behavioral therapy	Antihypertensive drugs	Jadad scale	Research analysis shows that acupuncture at GB20 (Fengchi) and Ll11 (Quchi) points has a definite curative effect and high safety in the treatment of essential hypertension.
Han et al., 2019 (China) [34]	Chinese	7 (722)	Acupuncture+Tianma Gouteng decoction	Antihypertensive drugs/Tianma Gouteng decoction	Cochrane criteria	Acupuncture combined with Tianma Gouteng decoction has a good curative effect in the treatment of hypertension.
Lee et al., 2009 (South Korea) [35]	English	11 (847)	Acupuncture or acupuncture+antihypertensive drugs	Placebo (sham acupuncture)/antihypertensive drugs/lifestyle intervention	Oxford scale	Considering the limitation of the four positive noninferiority studies and the results of the meta-analysis of the three sham-controlled studies, the notion that acupuncture may lower high blood pressure is inconclusive.
Li et al., 2014 (China) [36]	English	4 (386)	Acupuncture or acupuncture+antihypertensive drugs	Placebo (sham acupuncture)	Jadad scale	Our results are consistent with the conclusion that acupuncture significantly lowers blood pressure in patients taking antihypertensive medications.
Zhao et al., 2015 (China) [37]	English	23 (1788)	Acupuncture/acupuncture+lifestyle modification/abdominal acupuncture+antihypertensive drugs	Lifestyle modification/antihypertensive drugs/sham acupuncture	Cochrane criteria	Our review provided evidence of acupuncture as an adjunctive therapy to medication for treating hypertension, while the evidence for acupuncture alone lowering blood pressure is insufficient.
Chen et al., 2018 (China) [38]	English	30 (2107)	Acupuncture or electroacupuncture or + lifestyle modifications/or + antihypertensive drugs	Sham acupuncture or lifestyle modifications or antihypertensive drugs	Cochrane criteria	Our systematic review indicates there is inadequate high-quality evidence that acupuncture therapy is useful in treating hypertension, as the exact effect and safety of acupuncture therapy for hypertension are still unclear.
Zhang et al., 2022 (China) [39]	English	10 (1196)	Acupuncture therapy or + nonacupuncture hypertension treatment measures	No treatment/nonacupuncture hypertension treatment measures	Cochrane criteria	Existing evidence shows that acupuncture could be used for treating hypertension.

**Table 3:** Result of the AMSTAR-2 assessments.

Author, year	Q1	Q2	Q3	Q4	Q5	Q6	Q7	Q8	Q9	Q10	Q11	Q12	Q13	Q14	Q15	Q16	Quality
Liu et al., 2012 [26]	Y	N	N	PY	Y	Y	N	PY	PY	Y	Y	N	Y	Y	N	Y	VL
Yu et al., 2013 [27]	Y	N	N	PY	Y	Y	N	PY	PY	N	Y	N	Y	N	Y	N	VL
Zhang et al., 2013 [28]	Y	N	N	PY	Y	Y	N	Y	Y	Y	Y	N	Y	N	N	Y	VL
Qian, 2013 [29]	Y	N	N	PY	Y	Y	N	N	Y	N	Y	N	N	N	N	N	VL
Guo et al., 2013 [30]	Y	N	N	PY	Y	Y	N	N	Y	Y	Y	N	N	N	Y	Y	VL
Zhang et al., 2014 [31]	Y	N	N	PY	Y	Y	N	PY	PY	Y	Y	N	Y	Y	N	Y	VL
Zhang et al., 2017 [32]	Y	N	N	PY	Y	Y	N	PY	Y	Y	Y	N	Y	Y	Y	Y	VL
Zhu and Ding, 2018 [33]	N	N	N	PY	Y	Y	N	PY	PY	Y	Y	N	N	N	Y	Y	VL
Han et al., 2019 [34]	Y	N	N	PY	Y	Y	N	PY	Y	Y	Y	N	Y	Y	Y	Y	VL
Lee et al., 2009 [35]	Y	N	N	PY	Y	Y	N	PY	PY	Y	Y	N	Y	N	N	Y	VL
Li et al., 2014 [36]	Y	N	N	PY	Y	Y	N	Y	PY	Y	Y	N	N	N	N	Y	VL
Zhao et al., 2015 [37]	Y	N	N	PY	Y	Y	N	Y	Y	Y	Y	Y	Y	Y	N	Y	VL
Chen et al., 2018 [38]	Y	Y	N	PY	Y	Y	N	Y	Y	Y	Y	Y	Y	Y	Y	Y	L
Zhang et al., 2022 [39]	Y	Y	N	PY	Y	Y	N	PY	Y	Y	Y	N	Y	N	Y	Y	L

Note: Y: yes; PY: partial yes; N: no; VL: very low; L: low; H: high. Q2, Q4, Q7, Q9, Q11, Q13, and Q15 are key areas.

**Table 4:** Results of the ROBIS assessments.

Section/topic	Liu et al., 2012 (China) [26]	Yu et al., 2013 (China) [27]	Zhang et al., 2013 (China) [28]	Qian, 2013 (China) [29]	Guo et al., 2013 (China) [30]	Zhang et al., 2014 (China) [31]	Zhang et al., 2017 (China) [32]	Zhu and Ding, 2018 (China) [33]	Han et al., 2019 (China) [34]	Lee et al., 2009 (South Korea) [35]	Li et al., 2014 (China) [36]	Zhao et al., 2015 (China) [37]	Chen et al., 2018 (Chin a) [38]	Zhang et al., 2022 (China) [39]
Phase 1														
Assessing relevance	L	L	L	L	L	L	L	L	L	L	L	L	L	L
Patients/population(s)	Y	Y	Y	Y	Y	Y	Y	Y	Y	Y	Y	Y	Y	Y
Intervention(s)	Y	Y	Y	Y	Y	Y	Y	Y	Y	Y	Y	Y	Y	Y
Comparator(s)	Y	Y	Y	Y	Y	Y	Y	Y	Y	Y	Y	Y	Y	Y
Outcome(s)	Y	Y	Y	Y	Y	Y	Y	Y	Y	Y	Y	Y	Y	Y
Phase 2-domain 1: study eligibility criteria	L	L	L	L	L	L	L	L	L	L	L	L	L	L
2.1.1	Py	PY	PY	PY	PY	PY	PY	PY	PY	PY	PY	PY	Y	Y
2.1.2	Y	Y	Y	Y	Y	Y	Y	Y	Y	Y	Y	Y	Y	Y
2.1.3	Y	Y	Y	Y	Y	Y	Y	Y	Y	Y	Y	Y	Y	Y
2.1.4	Y	Y	Y	Y	Y	Y	Y	Y	Y	Y	Y	Y	Y	Y
2.1.5	Y	Y	Y	Y	Y	Y	Y	Y	Y	Y	Y	Y	Y	Y
Phase 2-domain 2: identification and selection of studies	H	H	H	H	H	H	H	H	H	H	L	L	L	L
2.2.1	Py	N	Y	N	N	N	N	N	PY	Y	PY	PY	PY	PY
2.2.2	Y	N	N	N	Y	N	N	N	N	Y	Y	Y	Y	Y
2.2.3	PN	PN	PN	PN	PN	PN	PN	PN	PN	PN	PY	Y	PY	Y
2.2.4	PY	Y	Y	Y	N	N	Y	Y	Y	Y	Y	Y	Y	Y
2.2.5	Y	Y	Y	Y	Y	Y	Y	Y	Y	Y	Y	Y	Y	Y
Phase 2-domain 3: collection and study appraisal	H	L	L	H	H	L	L	H	L	H	H	L	L	L
2.3.1	Y	Y	Y	Y	Y	Y	Y	Y	Y	Y	Y	Y	Y	Y
2.3.2	N	Y	Y	N	N	Y	Y	N	Y	Y	Y	Y	Y	Y
2.3.3	Y	Y	Y	Y	Y	Y	Y	Y	Y	Y	Y	Y	Y	Y
2.3.4	N	Y	Y	Y	Y	Y	Y	Y	Y	N	N	Y	Y	Y
2.3.5	Y	Y	Y	Y	Y	Y	Y	Y	Y	Y	Y	Y	Y	Y
Phase 2-domain 4: synthesis and findings	H	H	H	H	H	H	H	H	H	H	H	H	L	H
2.4.1	PN	PN	PN	PN	PN	PN	PN	PN	PN	PN	PN	PY	PY	PY
2.4.2	NI	NI	NI	NI	NI	NI	NI	NI	NI	NI	NI	NI	Y	Y
2.4.3	Y	Y	Y	Y	Y	Y	Y	Y	Y	Y	Y	Y	Y	Y
2.4.4	Y	N	N	N	N	Y	Y	N	Y	N	Y	Y	Y	N
2.4.5	NI	NI	N	NI	N	NI	N	N	NI	N	N	N	Y	N
2.4.6	N	N	N	N	N	Y	N	N	Y	N	Y	Y	Y	N
Phase 3 risk of bias in the review	H	H	H	H	H	H	H	H	H	H	H	H	H	H
A	N	N	N	N	N	N	N	N	N	N	N	N	Y	N
B	Y	Y	Y	Y	Y	Y	Y	Y	Y	Y	Y	Y	Y	Y
C	N	Y	Y	Y	N	Y	N	N	N	N	Y	N	N	N

Note:Y: yes; PY: probably yes; PN: probably no; N: no; NI: no information; L: low risk; H: high risk.

**Table 5:** Results of the PRISMA checklist.

Items	Liu et al., 2012 (China) [26]	Yu et al., 2013 (China) [27]	Zhang et al., 2013 (China) [28]	Qian, 2013 (China) [29]	Guo et al., 2013 (China) [30]	Zhang et al., 2014 (China) [31]	Zhang et al., 2017 (China) [32]	Zhu and Ding, 2018 (China) [33]	Han et al., 2019 (China) [34]	Lee et al., 2009 (South Korea) [35]	Li et al., 2014 (China) [36]	Zhao et al., 2015 (China) [37]	Chen et al., 2018 (China) [38]	Zhang et al., 2022 (China) [39]	Number of yes and partially yes(%)
Q1. Title	Y	N	N	Y	Y	Y	Y	Y	Y	Y	Y	Y	Y	Y	100%
Q2. Structured summary	PY	PY	PY	PY	PY	PY	PY	PY	PY	PY	PY	PY	PY	PY	100%
Q3. Rationale	Y	Y	Y	Y	PY	Y	Y	PY	Y	Y	Y	Y	Y	Y	100%
Q4. Objectives	PY	PY	PY	PY	PY	PY	PY	PY	PY	PY	Y	Y	Y	Y	100%
Q5. Protocol and registration	N	N	N	N	N	N	N	N	N	N	N	N	Y	Y	14%
Q6. Eligibility criteria	PY	Y	Y	Y	Y	Y	Y	PY	Y	Y	Y	Y	Y	Y	100%
Q7. Information sources	PY	PY	PY	PY	PY	PY	PY	PY	PY	Y	Y	Y	Y	Y	100%
Q8. Search	N	N	N	N	N	N	N	N	N	N	N	Y	PY	Y	21%
Q9. Study selection	N	PY	PY	N	N	PY	PY	N	PY	N	PY	PY	PY	PY	64%
Q10. Data collection process	PY	PY	PY	PY	PY	N	PY	N	N	PY	PY	PY	PY	PY	79%
Q11. Data items	N	PY	PY	N	PY	N	PY	N	N	PY	PY	PY	PY	PY	64%
Q12. Risk of bias in individual studies	PY	PY	PY	PY	PY	PY	PY	PY	PY	PY	PY	PY	PY	PY	100%
Q13. Summary measures	Y	Y	Y	Y	Y	Y	Y	Y	Y	Y	Y	Y	Y	Y	100%
Q14. Synthesis of results	Y	Y	Y	Y	Y	Y	Y	Y	Y	PY	Y	Y	Y	Y	100%
Q15. Risk of bias across studies	N	PY	N	N	N	N	PY	PY	N	N	Y	N	Y	Y	43%
Q16. Additional analyses	N	N	PY	N	N	N	PY	PY	N	N	Y	Y	Y	Y	50%
Q17. Study selection	PY	PY	PY	PY	PY	PY	Y	PY	PY	Y	Y	Y	Y	Y	100%
Q18. Study characteristics	PY	Y	Y	N	N	Y	Y	PY	Y	Y	Y	Y	Y	Y	86%
Q19. Risk of bias within studies	PY	PY	PY	PY	N	PY	PY	PY	PY	PY	PY	Y	Y	Y	93%
Q20. Results of individual studies	Y	Y	Y	PY	Y	Y	Y	Y	Y	Y	Y	Y	Y	Y	100%
Q21. Synthesis of results	PY	PY	PY	PY	PY	PY	Y	PY	PY	Y	Y	Y	Y	Y	100%
Q22. Risk of bias across studies	N	PY	N	N	PY	N	PY	PY	PY	N	N	N	Y	Y	50%
Q23. Additional analysis	N	N	PY	N	N	N	PY	PY	N	N	Y	Y	Y	Y	50%
Q24. Summary of evidence	N	N	N	N	N	N	N	N	N	N	N	N	N	N	0%
Q25. Limitations	PY	PY	PY	Y	N	Y	Y	PY	Y	PY	Y	Y	Y	Y	93%
Q26. Conclusions	Y	Y	Y	Y	Y	Y	Y	Y	Y	Y	Y	Y	Y	Y	100%
Q27. Funding	PY	N	PY	N	PY	PY	PY	PY	PY	PY	Y	Y	PY	Y	86%

Note: Y: yes; N: no; PY: partial yes.

**Table 6:** Results of evidence quality.

Author, year	Outcomes	Intervention vs. comparison	Studies (participants)	Limitations	Inconsistency	Indirectness	Imprecision	Publication bias	Relative effect (^∗^95% CI)	Heterogeneity	Quality
Liu et al., 2012 [26]	The effective rate of lowering blood pressure	Acupuncture vs. western medicine	7 (612)	-1①	0	0	-1③	0	OR = 0.93 (0.60, 1.45)	*I* ^2^ = 49%	L
		Acupuncture+western medicine vs. western medicine	3 (175)	-1①	0	0	-1③	-1⑤	OR = 2.95 (1.45, 6.01)	*I* ^2^ = 0%	VL
	The effective rate of improving symptoms	Acupuncture vs. western medicine	3 (180)	-1①	0	0	-1③	-1⑤	OR = 2.56 (1.22, 5.39)	*I* ^2^ = 0%	VL
		Acupuncture+ western medicine vs. western medicine	1 (60)	-1①	0	0	-2③	-1⑤	OR = 9.33, (1.87, 46.68)	*I* ^2^ = 0%	VL

Yu et al., 2013 [27]	The effective rate of lowering blood pressure	Acupuncture vs. western medicine	14 (1164)	-1①	-1②	0	0	0	RR = 1.036, (0.946, 1.135)	*I* ^2^ = 64.7%	L
	The efficacy of improving SBP		10 (768)	-1①	-1②	0	0	0	SMD = −0.12 (-0.378, 0.129)	*I* ^2^ = 66.4%	L
	The efficacy of improving DBP		10 (768)	-1①	0	0	0	0	SMD = −0.051 (-0.195, 0.092)	*I* ^2^ = 44.9%	M

Zhang et al., 2013 [28]	The effect of reducing the magnitude of SBP	Acupuncture vs. western medicine	8 (772)	-1①	-1②	0	-1③	0	MD = 1.35 (0.11, 2.59)	*I* ^2^ = 90%	VL
		Acupuncture+western medicine vs. western medicine	2 (140)	-1①	0	0	-1③	-1⑤	MD = 8.30 (5.51, 11.09)	*I* ^2^ = 13%	VL
		Acupuncture vs. sham acupuncture	1 (160)	-1①	-1②	0	-1③	-1⑤	MD = 7.00 (4.67, 9.33)	Not applicable	VL
	The effect of reducing the magnitude of DBP	Acupuncture vs. western medicine	8 (772)	-1①	-1②	0	-1③	0	MD = 0.52 (-1.43, 2.46)	*I* ^2^ = 78%	VL
		Acupuncture+western medicine vs. western medicine	2 (140)	-1①	0	0	-1③	-1⑤	MD = 4.66 (2.88, 6.45)	*I* ^2^ = 0%	VL
		Acupuncture *vs.* sham acupuncture	1 (160)	-1①	-1②	0	-1③	-1⑤	MD = 3.0 (1.29, 4.71)	Not applicable	VL

Qian, 2013 [29]	The efficacy of improving 24 h SBP	Acupuncture *vs.* sham acupuncture	1	-1①	-1②	0	-1③	-1⑤	MD = −5.55 (-8.72, -1.28)	No information	VL
		Acupuncture+traditional therapy(western medicine/lifestyle modification) vs. traditional therapy	2	-1①	-1②	0	-1③	-1⑤	MD = −7.51 (-10.37, -4.65)	No information	VL
	The efficacy of improving 24 h DBP	Acupuncture vs. sham acupuncture	1	-1①	-1②	0	-1③	-1⑤	No statistical significance	No information	VL
		Acupuncture+traditional therapy(western medicine/lifestyle modification) vs. traditional therapy	2	-1①	-1②	0	-1③	-1⑤	MD = −2.27 (-4.32, -0.22)	No information	VL
	The efficacy of improving 12 h SBP	Acupuncture vs. sham acupuncture	1	-1①	-1②	0	-1③	-1⑤	MD = −5.00 (-8.56, -1.44)	No information	VL
		Acupuncture+traditional therapy(western medicine/lifestyle modification) vs. traditional therapy	6	-1①	-1②	0	-1③	0	MD = −7.66 (-9.45, -5.86)	No information	VL
	The efficacy of improving 12 h DBP	Acupuncture vs. sham acupuncture	1	-1①	-1②	0	-1③	-1⑤	No statistical significance	No information	VL
		Acupuncture+traditional therapy(western medicine/lifestyle modification) vs. traditional therapy	6	-1①	-1②	0	-1③	0	MD = −2.87 (-4.16, -1.57)	No information	VL

Guo et al., 2013 [30]	The effective rate of lowering blood pressure	Acupuncture+western medicine vs. western medicine	10 (679)	-1①	0	0	-1③	0	OR = 5.23 (3.24, 8.44)	*I* ^2^ = 0%	L
	The efficacy of improving SBP		4 (275)	-1①	0	0	-1③	0	MD = −8.35 (-10.89, -5.81)	*I* ^2^ = 0%	L
	The efficacy of improving DBP		4 (275)	-1①	-1②	0	-1③	0	MD = −6.33 (-7.97, -4.69)	*I* ^2^ = 92%	VL

Zhang et al., 2014 [31]	The effective rate of lowering blood pressure	Acupuncture vs. western medicine	7 (612)	-1①	-1②	0	-1③	0	OR = 0.95 (0.45, 2.00)	*I* ^2^ = 55%	VL
		Acupuncture+western medicine vs. western medicine	4 (262)	-1①	0	0	-1③	0	OR = 5.13 (2.60, 10.11)	*I* ^2^ = 0%	L
	The efficacy of improving SBP	Acupuncture vs. western medicine	3 (180)	-1①	0	0	-1③	-1⑤	WMD = −3.26 (-7.98, 1.46)	*I* ^2^ = 0%	VL
		Acupuncture+western medicine vs. western medicine	2 (152)	-1①	0	0	-1③	-1⑤	WMD = −9.50 (-13.66, -5.34)	*I* ^2^ = 0%	VL
	The efficacy of improving DBP	Acupuncture vs. western medicine	3 (180)	-1①	0	0	-1③	-1⑤	WMD = −2.17 (-5.02, 0.68)	*I* ^2^ = 0%	VL
		Acupuncture+western medicine vs. western medicine	2 (152)	-1①	0	0	-1③	-1⑤	*WMD* = −0.16 (-2.52, 2.19)	*I* ^2^ = 0%	VL

Zhang L, 2017 [32]	The efficacy of improving SBP	Acupuncture vs. western medicine	23 (1705)	-1①	-1②	0	0	-1④	*SMD* = −0.66 (-1.03, -0.29)	*I* ^2^ = 92%	VL
		Acupuncture+western medicine vs. western medicine	11 (1029)	-1①	0	0	0	-1④	*SMD* = −1.14 (-1.31, -0.96)	*I* ^2^ = 38%	L
	The efficacy of improving DBP	Acupuncture vs. western medicine	23 (1705)	-1①	-1②	0	0	-1④	*SMD* = −0.61 (-1.02, -0.21)	*I* ^2^ = 93%	VL
		Acupuncture+western medicine vs. western medicine	11 (1029)	-1①	-1②	0	0	-1④	*SMD* = −1.10 (-1.63, -0.58)	*I* ^2^ = 93%	VL
	The effective rate of lowering blood pressure	Acupuncture vs. western medicine	28 (2271)	-1①	-1②	0	0	-1④	*RR* = 1.10 (1.03, 1.17)	*I* ^2^ = 69%	VL
		Acupuncture+western medicine vs. western medicine	14 (1125)	-1①	0	0	0	-1④	*RR* = 1.19 (1.13, 1.25)	*I* ^2^ = 5%	L
	The effective rate of improving symptoms	Acupuncture vs. western medicine	7 (465)	-1①	0	0	0	-1④	*RR* = 1.21 (1.11,1.31)	*I* ^2^ = 0%	L
		Acupuncture+western medicine *vs.* western medicine	3 (276)	-1①	0	0	0	-1④	*RR* = 1.19 (1.09,1.31)	*I* ^2^ = 12%	L
	The effective rate of comprehensive treatment	Acupuncture vs. western medicine	5 (394)	-1①	-1②	0	0	-1④	*RR* = 1.38 (1.14, 1.66)	*I* ^2^ = 61%	VL
		Acupuncture+western medicine vs. western medicine	2 (184)	-1①	0	0	0	-1④⑤	*RR* = 1.20 (1.07, 1.33)	*I* ^2^ = 0%	L

Zhu and Ding, 2018 [33]	The effective rate of comprehensive treatment	Acupuncture vs. western medicine/acupuncture+western medicine vs. western medicine/acupuncture+behavior therapy vs. western medicine	22 (1758)	-1①	0	0	-1③	0	OR = 2.49 (1.92, 4.24)	*I* ^2^ = 47%	L
	The efficacy of improving SBP		13 (908)	-1①	-1②	0	0	0	*WMD* = −4.50 (-6.45,-2.55)	*I* ^2^ = 86%	L
	The efficacy of improving DBP		13 (908)	-1①	-1②	0	0	0	*WMD* = −3.14 (-4.61, -1.66)	*I* ^2^ = 86%	L

Han et al., 2019 [34]	The effective rate of comprehensive treatment	Acupuncture+Tianma Gouteng decoction vs. western medicine/Tianma Gouteng decoction	6 (694)	-1①	0	0	-1③	-1④	OR = 5.39 (2.97, 9.80)	*I* ^2^ = 0%	VL
	The efficacy of improving SBP		3 (358)	-1①	-1②	0	-1③	-1④	OR = −15.49 (-18.48, -12.50)	*I* ^2^ = 53%	VL
	The efficacy of improving DBP		3 (358)	-1①	0	0	-1③	-1④	OR = −9.71 (-11.84, -7.57)	*I* ^2^ = 50%	VL

Lee et al., 2009 [35]	The effect of reducing the magnitude of SBP	Acupuncture vs. sham acupuncture	3 (358)	-1①	-1②	0	-1③	0	*MD* = −5 (-12, 1)	*I* ^2^ = 92%	VL
		Acupuncture+medication vs. sham acupuncture+western medicine	2 (170)	-1①	0	0	-1③	-1⑤	*MD* = −8 (-10, -5)	*I* ^2^ = 0%	VL
	The effect of reducing the magnitude of DBP	Acupuncture vs. sham acupuncture	3 (358)	-1①	-1②	0	-1③	0	*MD* = −3 (-6, 0)	*I* ^2^ = 79%	VL
		Acupuncture+medication vs. sham acupuncture+western medicine	2 (170)	-1①	0	0	-1③	-1⑤	*MD* = −4 (-6, -2)	*I* ^2^ = 0%	VL

Li DZ, 2014 [36]	The efficacy of improving SBP	Electroacupuncture/auricular acupuncture vs. sham acupuncture	2 (216)	-1①	0	0	-1③	0	*MD* = 1.33 (-2.50,5.16)	*I* ^2^ = 44%	L
		Electroacupuncture/auricular acupuncture or + antihypertensive drugs vs. sham acupuncture	2 (170)	-1①	0	0	-1③	-1⑤	*MD* = −8.58 (-10.13, -7.03)	*I* ^2^ = 17%	VL
	The efficacy of improving DBP	Electroacupuncture/auricular acupuncture vs. sham acupuncture	2 (216)	-1①	-1②	0	-1③	0	*MD* = −0.18 (-3.98, 3.62)	*I*2 = 63%	VL
		Electroacupuncture/auricular acupuncture or + antihypertensive drugs vs. sham acupuncture	2 (170)	-1①	0	0	0	-1⑤	*MD* = −4, 54 (-5.08, -4.00)	*I* ^2^ = 0%	L

Zhao et al., 2015 [37]	The efficacy of improving SBP	Acupuncture vs. western medicine	7 (510)	-1①	-1②	0	-1③	0	*MD* = −0.56 (-3.02, 1.89)	*I* ^2^ = 60%	VL
		Acupuncture+western medicine vs. western medicine	3 (170)	-1①	-1②	0	-1③	-1⑤	*MD* = −9.04 (-20.11, 2.02)	*I* ^2^ = 94%	VL
		Acupuncture+lifestyle modification vs. lifestyle modification	1 (60)	-1①	-1②	0	-1③	-1⑤	*MD* = −10.53 (-27.52, 6.46)	Not applicable	VL
	The efficacy of improving DBP	Acupuncture vs. western medicine	7 (510)	-1①	0	0	-1③	0	*MD* = −1.01 (-2.26, 0.24)	*I* ^2^ = 23%	L
		Acupuncture+western medicine vs. western medicine	3 (170)	-1①	-1②	0	-1③	-1⑤	*MD* = −2.87 (-8.45, 2.72)	*I* ^2^ = 86%	VL
		Acupuncture+lifestyle modification vs. lifestyle modification	1 (60)	-1①	-1②	0	-1③	-1⑤	*MD* = −7.52 (-15.06, 0.02)	Not applicable	VL
	The effect of reducing the magnitude of SBP	Acupuncture vs. sham acupuncture	2 (216)	-1①	0	0	0	0	*MD* = 0.30 (-0.27, 0.88)	*I* ^2^ = 0%	M
		Acupuncture+western medicine vs. sham acupuncture+western medicine	2 (170)	-1①	0	0	-1③	-1⑤	*MD* = −7.47 (-10.43, -4.51)	*I* ^2^ = 0%	VL
	The effect of reducing the magnitude of DBP	Acupuncture vs. sham acupuncture	2 (216)	-1①	0	0	-1③	0	*MD* = −1.40 (-2.37, -0.44)	*I* ^2^ = 8%	L
		Acupuncture+western medicine vs. sham acupuncture+western medicine	2 (170)	-1①	0	0	-1③	-1⑤	*MD* = −4.22 (-6.26, -2.18)	*I* ^2^ = 0%	VL

Chen et al., 2018 [38]	The effective rate of lowering blood pressure	Acupuncture vs. antihypertensive drugs	9 (517)	-1①	-1②	0	0	-1④	*RR* = 1.12 (0.98, 1.28)	*I* ^2^ = 78%	VL
		Acupuncture+lifestyle modifications vs. lifestyle modifications	2 (187)	-1①	0	0	0	-1④⑤	*RR* = 1.2 (1.05, 1.36)	*I* ^2^ = 0%	L
		Acupuncture+antihypertensive drugs vs. antihypertensive drugs	7 (517)	-1①	0	0	0	-1④	*RR* = 1.17 (1.08, 1.27)	*I* ^2^ = 0%	L
		Electro-acupuncture vs. antihypertensive drugs	2 (99)	-1①	0	0	0	-1④⑤	*RR* = 0.94 (0.76, 1.16)	*I* ^2^ = 0%	L
	The efficacy of improving SBP	Acupuncture vs. antihypertensive drugs	8 (541)	-1①	0	0	-1③	-1④	MD =1.4 (-1.32,4.12)	*I*2 = 57%	VL
		Acupuncture vs. no treatment	1 (30)	-1①	-1②	0	-1③	-1④⑤	*MD* = 5.2 (-2.99, 13.39)	Not applicable	VL
		Acupuncture vs. sham acupuncture	3 (106)	-1①	0	0	-1③	-1④⑤	*MD* = 1.59 (-4.63, 7.8)	*I* ^2^ = 65%	VL
		Acupuncture+lifestyle modifications vs. lifestyle modifications	3 (246)	-1①	-1②	0	-1③	-1④	*MD* = 10.38 (6.72, 14.04)	*I* ^2^ = 86%	VL
		Acupuncture+antihypertensive drugs vs. antihypertensive drugs	5 (365)	-1①	-1②	0	-1③	-1④	*MD* = 9.8 (2.95, 16.65)	*I* ^2^ = 94%	VL
		Acupuncture+antihypertesive drugs vs. sham acupuncture+antihypertensive drugs	2 (170)	-1①	0	0	-1③	-1④⑤	*MD* = 8.82 (5.1, 12.54)	*I* ^2^ = 35%	VL
		Electroacupuncture vs. antihypertensive drug	3 (200)	-1①	-1②	0	-1③	-1④	*MD* = 1.63 (-3.25, 6.52)	*I* ^2^ = 57%	VL
		Electroacupuncture+antihypertensive drugs vs. antihypertensive drugs	1 (59)	-1①	-1②	0	-1③	-1④⑤	*MD* = 9.12 (3.96, 14.28)	Not applicable	VL
	The efficacy of improving DBP	Acupuncture vs. antihypertensive drugs	8 (541)	-1①	-1②	0	-1③	-1④	*MD* = 2.04 (-0.59, 4.67)	*I* ^2^ = 83%	VL
		Acupuncture vs. no treatment	1 (30)	-1①	-1②	0	-1③	-1④⑤	*MD* = 6.1 (1.27,10.93)	Not applicable	VL
		Acupuncture vs. sham acupuncture	3 (106)	-1①	0	0	-1③	-1④⑤	*MD* = –0.01 (-2.59, 2.57)	*I* ^2^ = 15%	VL
		Acupuncture+lifestyle modifications vs. lifestyle modifications	3 (246)	-1①	-1②	0	-1③	-1④	*MD* = 5.74 (1.94, 9.54)	*I* ^2^ = 91%	VL
		Acupuncture+antihypertensive drugs vs. antihypertensive drugs	5 (365)	-1①	-1②	0	-1③	-1④	*MD* = 7.82 (4.67, 10.96)	*I* ^2^ = 79%	VL
		Acupuncture+antihypertensive drugs vs. sham acupuncture+antihypertensive drugs	2 (170)	-1①	0	0	-1③	-1④⑤	*MD* = 4.44 (1.7, 7.19)	*I* ^2^ = 36%	VL
		Electroacupuncture vs. antihypertensive drug	3 (200)	-1①	0	0	-1③	-1④	*MD* = −1.98 (-4.85, 0.62)	*I* ^2^ = 31%	VL
		Electroacupuncture+antihypertensive drugs vs. antihypertensive drugs	1 (59)	-1①	-1②	0	-1③	-1④⑤	*MD* = 4.46 (-0.25, 9.17)	Not applicable	VL

Zhang et al., 2022 [39]	The efficacy of improving SBP	Acupuncture/electroacupuncture/needle warming therapy vs. antihypertensive drugs	4 (1176)	-1①	0	0	0	-1④	*MD* = 3.62 (1.34, 5.90)	*I* ^2^ = 56%	L
	The efficacy of improving DBP		4 (1176)	1①	0	0	0	-1④	*MD* = 3.12 (1.03, 5.20)	*I* ^2^ = 77%	L

Note: VL: very low; L: low; M: moderate; H: high. ① The included studies have a large bias in methodology such as randomization, allocation concealment, and blinding. ② The confidence interval overlaps less or the *I*2 value of the combined results was larger. ③ The sample size from the included studies does not meet the optimal sample size or the confidence interval was not narrow enough. ④ The funnel chart is asymmetry. ⑤ Fewer studies were included, and their results were all positive, which may result in a large publication bias. ^∗^The 95% confidence interval does not cross the invalid line.

## Data Availability

The datasets analyzed during the current study are available from the corresponding author on reasonable request.

## Abbreviations

EH: Essential hypertension
CAD: Conventional antihypertensive drugs
TMGTD: Tianma Gouteng decoction
SBP: Systolic blood pressure
DBP: Diastolic blood pressure
SRs: Systematic reviews
MAs: Meta-analyses
RCTs: Randomized controlled trials
AMSTAR-2: A measurement tool to assess systematic reviews 2
ROBIS: Risk of bias in systematic reviews
PRISMA: Preferred reporting items for systematic reviews and meta-analyses
GRADE: Grading of recommendations assessment, development, and evaluation
TCM: Traditional Chinese medicine
CNKI: Chinese national knowledge infrastructure
CBM: Chinese biological medicine database.
